# Broad-Spectrum Antibacterial Peptide Kills Extracellular and Intracellular Bacteria Without Affecting Epithelialization

**DOI:** 10.3389/fmicb.2021.764451

**Published:** 2021-11-26

**Authors:** Anala Nepal, Synnøve Brandt Ræder, Caroline Krogh Søgaard, Maria Schei Haugan, Marit Otterlei

**Affiliations:** ^1^Department of Clinical and Molecular Medicine, Faculty of Medicine and Health Sciences, Norwegian University of Science and Technology (NTNU), Trondheim, Norway; ^2^Department of Medical Microbiology, St. Olav’s University Hospital, Trondheim, Norway

**Keywords:** APIM, antimicrobial resistance, β-clamp, translesion synthesis, antibacterial peptide, antimutagenic, ESKAPE

## Abstract

New antibacterial drugs with novel modes of action are urgently needed as antibiotic resistance in bacteria is increasing and spreading throughout the world. In this study, we aimed to explore the possibility of using APIM-peptides targeting the bacterial β-clamp for treatment of skin infections. We selected a lead peptide, named betatide, from five APIM-peptide candidates based on their antibacterial and antimutagenic activities in both G^+^ and G^–^ bacteria. Betatide was further tested in minimal inhibitory concentration (MIC) assays in ESKAPE pathogens, in *in vitro* infection models, and in a resistance development assay. We found that betatide is a broad-range antibacterial which obliterated extracellular bacterial growth of methicillin-resistant *Staphylococcus epidermidis* (MRSE) in cell co-cultures without affecting the epithelialization of HaCaT keratinocytes. Betatide also reduced the number of intracellular *Staphylococcus aureus* in infected HaCaT cells. Furthermore, long-time exposure to betatide at sub-MICs induced minimal or no increase in resistance development compared to ciprofloxacin and gentamicin or ampicillin in *S. aureus* and *Escherichia coli*. These properties support the potential of betatide for the treatment of topical skin infections.

## Introduction

Antibiotic resistance is a global problem. Widespread misuse of antibiotics, not only in human medicine but also in animal husbandry, has led to the emergence and spread of bacteria conferring resistance to multiple antibiotics. The World Health Organization (2017, 2018) has published a list of highly virulent bacteria with increasing multidrug resistance (MDR) such as the ESKAPE pathogens (*Enterococcus faecium*, *Staphylococcus aureus*, *Klebsiella pneumoniae*, *Acinetobacter baumannii*, *Pseudomonas aeruginosa*, and *Enterobacter* species). New antibiotics are urgently needed to cope with the increasing antimicrobial resistance (AMR) emerging in these pathogens because this is expected to give high annual global mortality and a high economic burden (World Health Organization, 2015).

Bacteria can become antibiotic resistant by harboring mutations in endogenous genes, or by taking up genes. This lack or gain of gene product may give them a functional advantage to resist the antibiotic. Cellular stress, for example, induced by antibiotic treatments, can activate the SOS damage response system (Beaber et al., 2004) and thereby DNA translesion synthesis (TLS) in bacteria (Pham et al., 2001; Goodman, 2002). TLS increases the mutation frequency and is the main cause of increased levels of endogenous mutations (Merrikh and Kohli, 2020). Targeting the SOS response is therefore a potential strategy for inhibiting mutagenesis and development of antibiotic resistance (Yakimov et al., 2021).

Antimicrobial peptides (AMPs) are one of the drug classes emerging as an alternative to conventional antibiotics. They usually act by targeting the bacterial cell wall but can also have intracellular targets. The major hurdle for AMP drug development has been low serum stability and toxicity (Magana et al., 2020). Another concern with AMPs is the development of cross-resistance, as prolonged bacterial exposure to one AMP in sublethal doses is shown to lead to resistance development to a wide variety of other AMPs; however, this is dependent on the nature of the peptides and their target(s) (Andersson et al., 2016).

APIM-peptides are cell-penetrating peptides (CPPs) containing the proliferating cell nuclear antigen (PCNA) interaction motif APIM, which were originally developed as anticancer drugs (Gilljam et al., 2009; Muller et al., 2013; Søgaard et al., 2018). Interestingly, they were found to have antibacterial properties in selected gram-positive (G^+^) and gram-negative (G^–^) bacteria (Nedal et al., 2020). This antibacterial property was mainly due to their ability to bind to the bacterial β-clamp via their APIM sequence, thereby inhibiting bacterial DNA replication and TLS. This killed the bacteria or, at sublethal concentrations, reduced their ability to develop resistance against other antibiotics if used in combination treatments (Nedal et al., 2020; Raeder et al., 2021). The APIM-peptide variant ATX-101, which is under development as an anticancer drug, was shown to have a favorable toxicity profile in a recent Phase I study (Lemech et al., 2021). Therefore, the two main concerns with AMPs, i.e., development of resistance and toxicity, may not apply to APIM-peptides.

Skin is the main physical barrier against bacteria. A bruise or an open cut after surgery makes the underlying tissue vulnerable to infection, and accordingly, use of topical antibiotics is shown to prevent infections and accelerate healing. However, the rise and spread of MDR bacteria has led to severe chronic infections in hospitalized patients where current antibiotics are ineffective (Filius and Gyssens, 2002). MDR variants of staphylococci are examples of bacteria that cause recurring infections in hospitalized patients. *Staphylococcus epidermidis* is a bacterium in the normal skin microbiota (Kloos and Musselwhite, 1975) and *S. aureus*, which is more virulent (Massey et al., 2006; Otto, 2009), is more common in the microbiota of the upper respiratory tract (Tulloch, 1954). Both species can become opportunistic pathogens post surgery, especially in immunocompromised patients and those with medical implants. *S. aureus* can in addition thrive intracellularly, making it hard to treat with antibiotics (Tuchscherr et al., 2011).

In wound healing, keratinocytes migrate toward the open gap after 24 h and protect the underlying cells before dermal layers take over and close the gap (Rousselle et al., 2019). In order to develop a drug for topical application, it is important that the reepithelialization capacity of the keratinocytes surrounding the wound area is not severely affected (Pastar et al., 2014). In this study, we selected a lead APIM-peptide, betatide, and examined its antibacterial potential and its effects on epithelialization of keratinocytes in two different cell line-based *in vitro* infection assays. We also examined the ability of bacteria to develop resistance against betatide and betatide’s activity on resistant and reference ESKAPE pathogens, alone and in combination with selected antibiotics.

## Materials and Methods

### Bacterial Strains

All bacterial strains used in this study are listed in Table 1. The reference strains are indicated by their ATCC and CCUG numbers, while the clinical strains, which were obtained from the strain collection at the Department of Medical Microbiology, St. Olav’s (SO) University Hospital, are indicated by their SO codes.

**TABLE 1:** Bacterial strains used in this study.

Bacterial species	Strain	Antibiotic resistance	Used in experiment
*Staphylococcus epidermidis*	SO-SEP9-1	Erythromycin, penicillin, cloxacillin/dicloxacillin (MRSE)	Epithelialization assay
*Staphylococcus aureus*	ATCC 29213	None	Intracellular infection assay, resistance assay
	SO-SAU19-1 (MRSA), SO-SAU19-2, -3 and -4 (FR-MRSA)	*mecA+*	MIC (ESKAPE), resistance assay
*Escherichia coli*	K-12 MG1655	None	MIC (ESKAPE), growth inhibition, mutagenesis and resistance assays
	SO-ECO19-1	ESBL-CARB-A(CTX-M-24)/D (OXA-48)	MIC (ESKAPE)
*Enterococcus faecium*	CCUG 59167	*vanA*+ (VRE)	MIC (ESKAPE)
	SO-EFU19-1	*vanB*+ (VRE)	MIC (ESKAPE)
*Enterococcus faecalis*	ATCC 29212	None	MIC (ESKAPE)
	SO-EFA19-1	*optrA*+	MIC (ESKAPE)
*Klebsiella pneumoniae*	ATCC 13883	None	MIC (ESKAPE)
	SO-KPN19-1	ESBL-CARBA-D (OXA-48-like)	MIC (ESKAPE)
*Pseudomonas aeruginosa*	ATCC 27853	None	MIC (ESKAPE)
	SO-PAE19-1	Multidrug resistant	MIC (ESKAPE)
*Acinetobacter baumannii*	ATCC 19606	None	MIC (ESKAPE)
	SO-ABA19-1	ESBL-CARBA-D (OXA-24)	MIC (ESKAPE)
*Enterobacter cloacae*	SO-ECL18-1	ESBL-CARBA (NDM)	MIC (ESKAPE)

### Antibiotic Resistance

For the clinical strains, this was essentially done as defined by EUCAST Clinical Breakpoints and guidance (EUCAST, 2021).

### APIM-Peptides

APIM-peptides (Innovagen, SE) used in this study have the same N-termini but differ in the composition of linkers and/or CPPs as shown in Table 2. Peptides 1 (RWLVK) and 2 (RWLVK*) are previously used in Nedal et al. (2020). A C-terminus FAM-labeled betatide (Innovagen) was used to study intracellular import. All the concentrations of APIM-peptides given in the different figures are net peptide concentrations, and 4 μg/ml equals approximately 1 μM.

**TABLE 2:** Properties of APIM-peptide variants.

N-termini*	Linker	CPP*	MIC (μg/ml)		Reduction of viability		Reduction of mutation frequency
MDRWLVK			*S. epidermidis* MRSE	*E. coli*	*E. coli* (60 μg/ml)	HEK293 (24 μg/ml)	*E. coli* (20 μg/ml)
^#^Peptide 1	W-KKKRK-I	R11	32	32	–	+	+
^#^Peptide 2/betatide	GILQ-WRK-I	R11	16	16	++++	+	++++
Peptide 3	GILQ-WRK-I	R10	16	32	ND	+	++
Peptide 4	GILQ-WRK-I	R9	16	32	++	ND	ND
Peptide 5	GILQ-WRK-I	R8	16	16	++	+	++

*MIC, Minimum inhibitory concentration.*

*All peptides were acetylated on the N-termini and amidated on the C-termini.

#Peptides 1 and 2 are named RWLVK and RWLVK*, respectively, in Nedal et al. (2020).

*“++” to “++++” denotes degree of reduced viability and mutation frequency relative to untreated control; “–” no effect; “+” tendency, but not a significant reduction; ND, not done. The raw data are shown in Supplementary Figures 1–3.*

### Cell Culture and Maintenance

HaCaT, a human spontaneous immortalized keratinocyte cell line, was cultured in Dulbecco’s Modified Eagle Medium (DMEM; 4.5 g/L glucose; Sigma-Aldrich), supplemented with 10% fetal bovine serum (FBS; Sigma-Aldrich) and 1 mM L-glutamine (Sigma-Aldrich). HEK293, an immortalized embryonic kidney cell line, was grown in DMEM (BioWhittaker, Walkersville, MD, United States) with the same supplements as described above. In addition, Fungizone^®^ amphotericin B (2.5 μg/ml; Gibco, Thermo Fisher Scientific, Waltham, MA, United States) and 1 mM antibiotic mixture containing 100 μg/ml penicillin and 100 μg/ml Streptomycin (Gibco) were added to the growth media. The cells were incubated at 37°C in a humidified incubator with 5% CO_2_.

### Minimal Inhibitory Concentration Assay

Minimal inhibitory concentration assay was conducted as recommended by the Clinical and Laboratory Standards Institute (CLSI) (Cockerill et al., 2012), similar to a previous report (Nedal et al., 2020). Briefly, bacterial colonies from blood agar plates were suspended and grown in Cation-Adjusted Mueller-Hinton Broth (CAMHB, 22.5 mg/ml Ca^2+^, 11 mg/ml Mg^2+^). The bacterial suspension was adjusted to 0.5 McFarland standard (∼1 × 10^8^ colony-forming units (CFU)/ml) and serial diluted 1:200 in CAMHB (∼5 × 10^5^ CFU/mL). This was subsequently added to polypropylene microtiter plates (Greiner, 100 μl/well, ∼5 × 10^4^ CFU/well) already prepared with betatide and different antibiotics as single agents or in combinations (11 μl/well, twofold serial dilutions). The suspension was plated out on blood agar plates to confirm the CFU/ml. Both the microtiter plates and the blood agar plates were incubated at 37°C for 24 h. The lowest concentration of antibiotics and/or betatide capable of inhibiting visible bacterial growth was determined as the MIC.

The MICs of ampicillin (Sigma, A9518), cefoxitin (Sigma, C4786), cefotaxime (Sigma, 219504), ceftazidime (Sigma, C3809), ceftriaxone (Sigma, C5793), ciprofloxacin (Sigma, 17850), clindamycin (Sigma, C5269), ertapenem (Sigma, SML1238), gentamicin (Gibco, 1510049), linezolid (Sigma, PZ0014), meropenem (Sigma, M2574), methicillin (Sigma, 51454), and fusidic acid (MedChemExpress, HY-B1350A) were determined in addition to that of betatide.

### Growth Inhibition Assay

An overnight culture of *Escherichia coli* was diluted 1:100 in Luria-Bertani (LB) medium and allowed to grow until an optical density (OD) at 600 nm (OD600) of 0.06–0.1. APIM-peptides were prepared by serial dilution in Milli-Q H_2_O and kept at 4°C. Fresh LB medium (60 μl) was added to a flat-bottom microtiter plate. The bacterial suspension was diluted 1:100 in LB, and 75 μl of this suspension was added to the wells. Finally, 15 μl of APIM-peptide solution (to final concentrations 60, 120, and 240 μg/ml) or distilled water (positive control) was added to the wells, reaching a total volume of 150 μl per well. Data are presented only for the dose that separated the effect of the different peptides, i.e., 60 μg/ml. A blank sterile medium was used as negative control. The plates were incubated with shaking at 510 rpm at 37°C inside a plate reader (TECAN, Spark^®^), and OD was read every hour over a period of 24 h.

The MIC for *E. coli* in LB medium is higher than that in CAMHB; thus, concentrations higher than the MIC given in Table 2 are used in the growth inhibition and mutagenesis assays.

### Viability

HEK293 cells (6,000 cells/well) were seeded in 96-well microtiter plates. After 4 h, APIM-peptides (12–48 μg/ml) were added, and the cells were incubated without change of media for up to 4 days. Viability was measured using the 3-(4,5-dimethylthiazol-2-yl)-2,5-diphenyltetrazolium bromide (MTT) assay as described (Gilljam et al., 2009). Data show the percentage of viable cells relative to untreated cells for 24 μg/ml at 72 h.

### Mutagenesis Assay

The rifampicin (Rif^R^) mutagenesis assay was performed as described (Nedal et al., 2020). Briefly, an overnight culture of *E. coli* was diluted 1:1,000 and grown until an OD600 of 0.01. APIM-peptides (20 μg/ml) were added to LB media with glass beads (for uniform distribution of APIM-peptides) and incubated for 30 min at 37°C. The pellets were next collected, resuspended in 500 μl PBS, and exposed to UV-C (20 mJ/cm^2^) in a six-well plate at 4°C. The unexposed bacteria (-UV) were otherwise handled exactly like the UV-exposed bacteria. Next, the cells were resuspended in LB media and incubated in a rotary shaker at 37°C at 250 rpm for 2 h before being harvested, diluted, and plated on LB with soft agar with and without rifampicin (100 μg/ml). Mutation frequency, Rif^R^/10^8^, is obtained as follows: (number of colonies on the rifampicin plate (Rif^R^)/(number of colonies on LB plates without antibiotics), per milliliter of bacterial suspension.

### Epithelialization Assay

The epithelialization assay is a modified version of the scratch test (Longaker et al., 1989; Walter et al., 2010). Briefly, HaCaT cells (1 × 10^6^ cells/well) were seeded in six-well plates with Steri-Strips™ (R1540, 3M Healthcare, United States) attached to the bottom. The cells were confluent in monolayer after 24 h (day 1), and the strips were then removed, creating even 3-mm gaps in the middle of the wells. The wells were washed 2× with PBS before fresh medium was added. The cells were next infected with 450 CFU/ml of an antibiotic-resistant *S. epidermidis* strain (MRSE, see Table 1) and treated with betatide (2–24 μg/ml). All treatments were done at day 1, and the epithelialization of the gaps was examined over a period of 7 days by taking pictures every 24 h in light microscopy (EVOS^®^ FL, Life Technologies). Bacterial growth was examined by plating of supernatants. The effect of betatide on noninfected HaCaT cells with regard to viability and epithelization was examined in parallel wells without MRSE.

Epithelialization was calculated from the change in the area of the gaps over time using freehand or rectangular selections in the software Fiji ImageJ 1.52p (National Institutes of Health, United States).

%Epithelialization

=(Area⁢of⁢gap⁢in⁢Day⁢ 1-Area⁢of⁢gap⁢in⁢each⁢consecutive⁢day)Area⁢of⁢gap⁢in⁢Day⁢ 1

The MIC for MRSE in DMEM (0.25 μg/ml) is lower than the MIC given in Table 2; thus, concentrations lower than MIC were used.

### Intracellular Infection Model

The intracellular infection model used was modified from Iqbal et al. (2016) by optimizing the number of multiplicity of infection (MOI: number of bacteria per cell) and time of infection. Briefly, HaCaT cells (6.5 × 10^4^/well) were seeded in 24-well plates and incubated overnight in a medium without antibiotics. The next day, approximately 1.25 × 10^5^ cells/well were infected with *S. aureus* at an MOI of 100 in antibiotic-free media. The plates were incubated for 3 h. Next, the cells were washed 2× with 500 μl PBS and treated with 100× MIC of gentamicin (100 μg/ml, MIC = 1 μg/ml) for 1 h, before further incubation in media with 10× MIC of gentamicin to kill the extracellular bacteria. Betatide (8–48 μg/ml) was added to the infected cells, and an equal amount of distilled water was added to the untreated control. After 16 h of incubation, the extracellular media (100 μl) from each well were plated to confirm the eradication of extracellular *S. aureus*. Next, the cells were washed 2× with 500 μl PBS before they were lysed with 0.2% Triton-X (500 μl) for 30 min at room temperature. The lysed samples were placed on ice, and 500 μl cold Tryptic Soy Broth was added before they were plated on blood agar plates. Data are presented for the dose that showed the best intracellular effects and low HaCaT cell toxicity, i.e., 24 μg/ml.

### Imaging

Intracellular import of betatide in HaCaT cells was examined using a fluorescent-tagged betatide (betatide-FAM, ∼20 μg/ml). Vybrant^®^ DyeCycle™ Ruby stain (VDR, 5 μM, V10273, Life Technologies™), which can penetrate plasma membranes, was used to stain DNA of live cells. Both betatide-FAM and VDR were added to live HaCaT cells immediately before (<2 min) examination in a Zeiss LSM 510 Meta laser scanning microscope equipped with a plan-apochromat × 63/1.4 oil immersion objective. FAM was excited at λ = 514 nm and detected above 515 nm, and VDR was excited at λ = 633 nm and detected above 650 nm. We used consecutive scans, and the optical slices were 1 μm.

### Resistance Development Assay

Bacteria (*E. coli* K-12 MG1655 and *S. aureus* ATCC 29213 and SO-SAU19-1) were serial passaged in CAMHB as described by Silverman et al. (2001) with some small modifications. Briefly, bacteria were passaged for up to 32 days in a round-bottom polypropylene microtiter plate (Greiner) in media containing 0.25, 0.5, 1, and 2× MIC of betatide or other antibiotics. In *E. coli*, the MIC was 0.06 μg/ml for ciprofloxacin and 16 μg/ml for ampicillin. In *S. aureus*, the MIC was 1 μg/ml for gentamicin, 0.25 μg/ml for ciprofloxacin in ATCC 29213, and 0.5 μg/ml for SO-SAU19-1. For every passage (each day), the new MIC was determined, and bacterial cells from the 0.5× MIC culture were continued for passage by adjusting this culture to ∼0.5 × 10^6^ CFU/ml in CAMHB. Fresh preparations of betatide/other antibiotics (0.25–2× MIC) were finally added to the diluted cultures, adjusted to the new MIC.

## Results

### Selection of the Most Efficient Antibacterial and Antimutagenic APIM-Peptide

The antibacterial effect of APIM-peptides was previously shown to be partly caused by the CPP part, although MIC was 2×–3× higher for the CPP only than for the full-length APIM-peptide variants (Nedal et al., 2020). We have also previously found that an APIM sequence linked to a CPP containing 11 arginines (R11) had higher antibacterial activity than the same sequence linked to HIV-TAT, transportan, and penetratin CPPs (data not shown). Peptide 1, which is based on the sequence of the original anticancer peptide (Muller et al., 2013), is previously shown to have lower antibacterial efficacy (higher MIC) than the same peptide with a different linker, peptide 2 (Nedal et al., 2020), and this was verified here (Table 2). The number of arginines (Rs) required to facilitate uptake into the nucleus of mammalian cells has been found to be eight, while an increased proportion of the peptide was detected in the cell membrane when Rs were increased to 16 (Futaki et al., 2001). Here, we therefore explored if the number of Rs in the CPP domain of peptide 2 affected the growth of bacteria and human cells differently. Table 2 includes comparison of performance of the peptides in more assays than previously reported (Nedal et al., 2020); therefore, we also included peptide 1 in these tests.

Reduction in the number of Rs from 11 to 8 (peptides 2–5) did not affect MIC in the MRSE strain, while peptides 2 and 5 had the lowest MIC in *E. coli* (K-12 MG1655) (Table 2). The MIC assay determines visual growth inhibition after 24 h; therefore, to explore potential differences in antibacterial efficacy in more detail, we examined these peptides’ inhibitory effect on the growth of *E. coli* over 24 h (for growth curves, see Supplementary Figure 1). We found that peptide 2 inhibited bacterial growth more than peptide 5 did in this assay (Table 2, reduction of viability, *E. coli*); thus, the superior antibacterial efficacy based on these two assays was determined to be that of peptide 2.

One of the most important factors to consider when selecting and developing a drug is low toxicity for human cells. The ideal situation would be to develop an APIM-peptide variant with a lower effect on mammalian cells and a larger effect on bacteria, i.e., to increase the therapeutics window. However, when the viability of HEK293 cells was tested after treatment with the peptides using the MTT assay, the peptides reduced the viability of HEK293 cells similarly, with peptide 5 (R8) possibly inhibiting the viability slightly more than the other peptide variants did (Table 2 and Supplementary Figure 2).

Peptides 1 and 2 are previously shown to inhibit TLS at sub-MICs via inhibition of polymerase V (Pol V) binding to the β-clamp (Nedal et al., 2020). Because inhibition of TLS is an important trait of these peptides, we compared these two peptides with the peptides with shorter CPPs for their ability to reduce the mutation frequency in *E. coli* using the Rif^R^ assay. We found that peptide 2 reduced the mutation frequency more efficiently than the other peptides did (Table 2 and Supplementary Figure 3).

In summary, these results show that the peptide with the GILQ-WRK-I linker and the R11 CPP is superior to the peptide with the W-KKKRK-I linker and to peptides with shorter arginine chains, with regard to antibacterial and antimutagenic properties, while the toxic effects on human cells are similar in all the peptide variants tested. Based on the results summarized in Table 2, peptide 2 was selected as the lead antibacterial peptide candidate and hereafter named betatide (beta-clamp targeting peptide).

### Betatide Has Low Minimal Inhibitory Concentration for *Enterococcus faecium*, *Staphylococcus aureus*, *Klebsiella pneumoniae*, *Acinetobacter baumannii*, *Pseudomonas aeruginosa*, and *Enterobacter cloacae* and Shows No Cross-Resistance With Other Antibiotics

Next, the activity of betatide against a wider selection of bacterial species from the ESKAPE list, i.e., MDR clinical isolates and corresponding reference strains, was examined (Table 3A). MICs for conventional antibiotics in the different MDR strains were determined in parallel with betatide, and this showed that the MDR strains had a 4× to >16,000× increase in MIC relative to their reference strains. However, betatide had an overall low MIC for all species (8–16 μg/ml) and was equally efficient against the reference strains as the clinical MDR isolates (except in one case: *Enterococcus faecalis*, 2× MIC). These results show that betatide has broad antibacterial activity and has no cross-resistance with the other antibiotics tested. This was expected as betatide has a ubiquitous bacterial target that is not shared by the other antibiotics. In some strains, a 2×–4× additive effect of the commercial antibiotic was detected when combined with 0.5× MIC of betatide, and an additive effect was observed more often for the MDR clinical isolates than for the reference strains (Table 3A).

**TABLE 3:** MIC values and combination effects of betatide and commercial antibiotics in **(A)** ESKAPE strains and **(B)** *Staphylococcus aureus* fusidic acid sensitive (MRSA) and resistant (FR-MRSA).

A	Reference strain		Clinical isolate/resistant strain	
	MIC (μg/ml)	Combination effect with 0.5× MIC betatide	MIC (μg/ml)	Combination effect with 0.5× MIC betatide
***E. faecium***	CCUG 59167 (*vanA*+)		SO-EFU19-1 (*vanB*+)	
Betatide	8		8	
Ampicillin	1	None	2,048	None
***S. aureus***	ATCC 29213		SO-SAU19-1 (MRSA)	
Betatide	16		16	
Methicillin	1	Additive 2×	8	Additive 4×
Cefoxitin	4	None	32	Additive 2×
Clindamycin	0.25	Additive 2×	2,048	Additive 2×
***K. pneumoniae***	ATCC 13883		SO-KPN19-1 (ESBL-CARBA-D)	
Betatide	16		16	
Gentamicin	0.50	Additive 4×	64	Additive 4×
Ertapenem	0.03	Additive 4×	32	Additive 2×
Cefotaxime	0.13	Additive 2×	2,048	None
***A. baumannii***	ATCC 19606		SO-ABA19-1 (ESBL-CARBA-D)	
Betatide	8		8	
Gentamicin	8	None	>1,000	NA
Meropenem	4	None	512	None
Ciprofloxacin	1	None	512	Additive 2×
***P. aeruginosa***	ATCC 27853		SO-PAE19-1 (multidrug resistant)	
Betatide	16		16	
Gentamicin	2	None	16	None
Meropenem	0.5	None	64	Additive 2×
Ceftazidime	2	None	128	None
***E. cloacae***			SO-ECL18-1 [ESBL-CARBA (NDM)]	
Betatide			16	
Gentamicin			>1,000	NA
Cefotaxime			>1,024	NA
Ceftazidime			>1,000	NA
***E. coli***	MG1655		SO-ECO19-1 (ESBL-CARB-A(CTX-M-24)/D(OXA-48))	
Betatide	16		16	
Gentamicin	0.5	None	>500	NA
Ertapenem	0.06	None	64	Additive 2×
Ceftriaxone	0.06	None	1,024	Additive 4×
***E. faecalis***	ATCC 29212		SO-EFA19-1 (*optrA+*)	
Betatide	8		16	
Linezolid	2	None	8	None

**B**				

***S. aureus***	ATCC 29213		SO-SAU19-1 (MRSA)	
Betatide	16		16	
Fusidic acid	0.25	Additive, 2×	0.25	Additive 8×
			SO-SAU 19-2 (FR-MRSA)	
Betatide			32	
Fusidic acid			8	Additive 2×
			SO-SAU 19-3 (FR-MRSA)	
Betatide			32	
Fusidic acid			8	Additive 4×
			SO-SAU 19-4 (FR-MRSA)	
Betatide			32	
Fusidic acid			8	Additive 2×

*NA, Not applicable; Combination effect = Additive effects, not (reduced MIC) of antibiotics when combining treatments with 0.5× MIC betatide. MIC values were confirmed in three independent experiments.*

Because fusidic acid is commonly used in the treatment of wound infections, we examined if betatide enhanced the effect of fusidic acid against *S. aureus*. An 8× reduction in MIC of fusidic acid was observed when combined with 0.5× MIC betatide in a fusidic acid-sensitive MRSA strain (Table 3B). Further, a 2×–4× additive effect was detected in three fusidic acid-resistant MRSA strains (FR-MRSA).

Altogether, these results support the potential of betatide, both as a single antibacterial agent and in combination with commonly used antibiotics.

### Betatide Eradicates Methicillin-Resistant *Staphylococcus epidermidis* Infections Without Affecting Epithelialization

**FIGURE 1:**
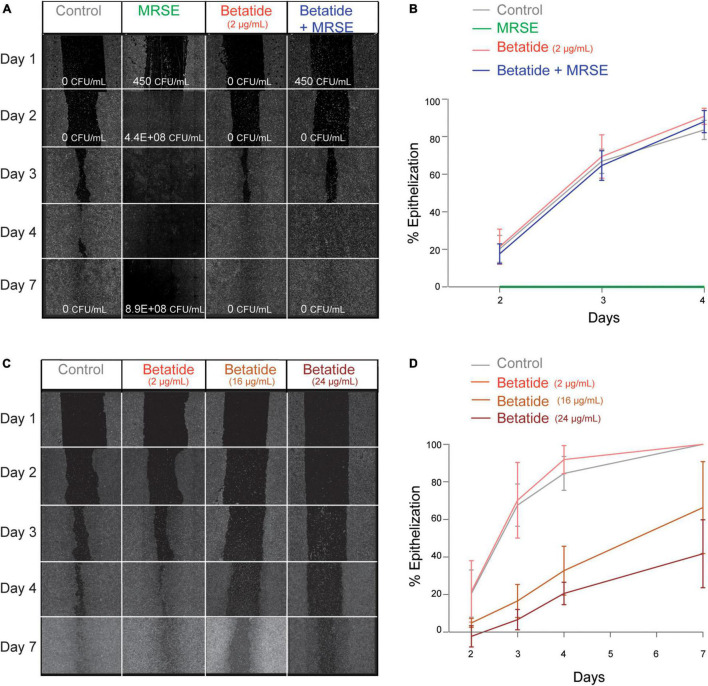
Betatide kills methicillin-resistant *Staphylococcus epidermidis* SO-SEP9-1 (MRSE) *in vitro*, without affecting the epithelialization of HaCaT keratinocytes. **(A)** Images from one representative biological replicate showing epithelialization in gaps on days 1–7 (day 1 = day of infection, day 2 = 24 h after infection) in the individual treatment groups: untreated cells (control, gray, first panel); cells infected with MRSE (green, second panel); cells treated with betatide (2 μg/ml, red, third panel); and cells infected with MRSE but treated with betatide (2 μg/ml, blue, fourth panel). Number of bacteria added on day 1 and obtained after plating of the supernatants on day 2 and day 7 is given as CFU/ml in the images. **(B)** Quantification of percentage of epithelialization in the gaps relative to the original area of the gaps in **(A)** and in two additional biological replicates, on days 1–4. Mean ± SD. **(C)** Images from one representative biological replicate showing levels of epithelialization with increasing doses of betatide. Control (gray, first panel) and betatide 2 μg/ml (red), 16 μg/ml (orange), and 24 μg/ml (dark red) in the second to fourth panels, respectively. **(D)** Quantification of the percentage of epithelialization in the gaps relative to the original area of the gaps in **(C)** and in two additional biological replicates on days 1–7. Mean ± SD.

In a mouse MRSA wound infection model, a gel containing a variant of the APIM-peptide significantly reduced the bacterial load with no visible toxicity on the skin (Nedal et al., 2020). As wound infections could be a suitable indication for these peptides and mouse skin may differ from human skin, we next more closely examined the efficacy of betatide and its effect on the epithelialization in an *in vitro* wound infection model. For this, we developed an epithelialization assay that is similar to the scratch test (Longaker et al., 1989; Walter et al., 2010), but where the gaps were made identical for accurate quantification by using strips. HaCaT cells infected with MRSE without treatment were all dead by day 2 after an exponential growth of the bacteria (Figure 1A, second panel). However, betatide (2 μg/ml) already eradicated MRSE from the culture wells at day 2 (Figure 1A, fourth panel). The epithelialization was completed at day 4, similar to the uninfected cells (Figure 1A, first and third panels). Thus, the epithelialization capacity of the cells was not affected at doses that completely abolished infection (epithelialization quantified in Figure 1B, CFU/ml depicted on the image in Figure 1A). The cells were cultured for up to 7 days without the infection re-emerging (Figure 1A, day 7, fourth and first panels).

When examining how epithelialization was affected by higher doses of betatide, a gradual decrease in percentage of epithelialization with increasing doses of betatide was observed (Figure 1C, quantified in Figure 1D). An approximately 70% decrease in epithelialization at day 4 was detected when using a betatide dose that was 12× higher than what is needed for a total eradication of the bacteria (24 μg/ml); however, the cells were not dying, and the epithelialization was re-established on day 7. Epithelialization was also re-established on day 7 after treatment with up to 40 μg/ml betatide (data not shown). Overall, these data indicate that doses that are more than 12× higher than the antibacterial dose could be used without severely affecting epithelialization.

### Betatide Reduces Intracellular *S. aureus* Infections

**FIGURE 2:**
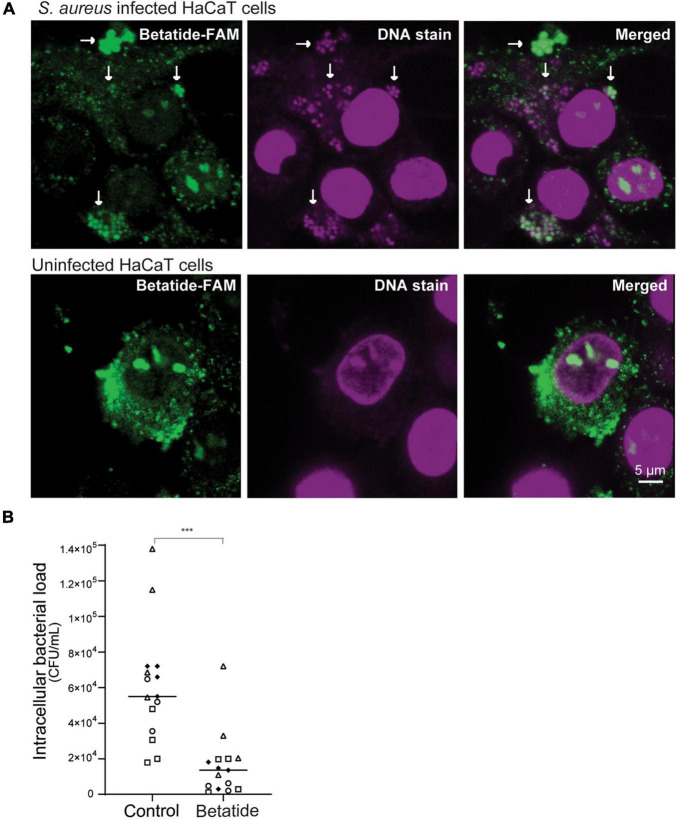
Betatide reduces intracellular bacterial load in HaCaT cells infected with *Staphylococcus aureus*. **(A)** Images show import of fluorescently tagged betatide (betatide-FAM, 20 μg/ml, green) in *S. aureus* ATCC 29213-infected HaCaT cells (upper panels) and uninfected HaCaT cells (lower panels). DNA is stained with Vybrant^®^ DyeCycle™ Ruby stain (VDR, 5 μM). Examples of small circular dots in the cytosol containing both betatide-FAM and DNA are marked with white arrows. **(B)** Intracellular bacterial load (CFU/ml) after *S. aureus* infection in HaCaT cells treated with betatide (24 μg/ml for 16 h) compared to untreated control. Extracellular *S. aureus* were eradicated by gentamicin treatment prior to this exposure in both the control and betatide-treated samples. Technical replicates from each biological replicate are shown with identical symbols (circles, triangles, squares, and diamonds). ***p < 0.0001 in unpaired two-tailed *t*-test with Welch’s correction.

Betatide is a variant of the APIM-peptide ATX-101, which is previously shown to be rapidly imported into multiple cells and to be distributed to all tissues upon intravenous infusion (Muller et al., 2013; Søgaard et al., 2018). Here, we show that fluorescent-labeled betatide (betatide-FAM, green) is rapidly taken up by live HaCaT cells (Figure 2A) and has similar subcellular localizations as ATX-101 (Muller et al., 2013); i.e., it is found in the cytosol, in the nuclei, and in the nucleoli. In addition, betatide-FAM in *S. aureus*-infected cells is found in small circular dots in the cytosol (highlighted by white arrows in Figure 2A, upper panel) that also are stained with live-cell DNA staining (magenta). These dots are not detected in uninfected HaCaT cells stained with live-cell DNA staining (lower panel), suggesting that these circular dots represent *S. aureus*.

To examine if betatide, which co-localizes with intracellular *S. aureus* (Figure 2A, merged image, white arrows), has antibacterial activity against intracellular *S. aureus*, we next measured intracellular bacterial counts in infected HaCaT cells treated with the peptide. Because *S. aureus* produces toxin that kills mammalian cells (Fraunholz and Sinha, 2012), optimization of the infection period and the number of infecting bacteria per cell was vital. We found that 100 MOI and infection for 3 h gave an intracellular infection without severe HaCaT cell cytotoxicity. The remaining extracellular bacteria were killed by gentamicin treatment prior to treatment of the infected cells with betatide (confirmed by plating at the time of harvest of the cells). A 4× reduction in intracellular bacterial load was found in betatide-treated cells (24 μg/ml), compared to untreated control (Figure 2B). Treatment with lower concentrations of betatide did not cause a significant reduction in CFU, while higher concentrations killed the infected HaCaT cells. Toxins from *S. aureus* likely sensitized the infected cells as uninfected HaCaT cells tolerated up to 40 μg/ml betatide. The maximum tolerated dose of betatide may therefore be different in different types of bacterial infections. In conclusion, these results show that betatide is rapidly taken up in mammalian cells where it retains its antibacterial activity.

### Bacteria Have Low Capacity to Develop Resistance Against Betatide

**FIGURE 3:**
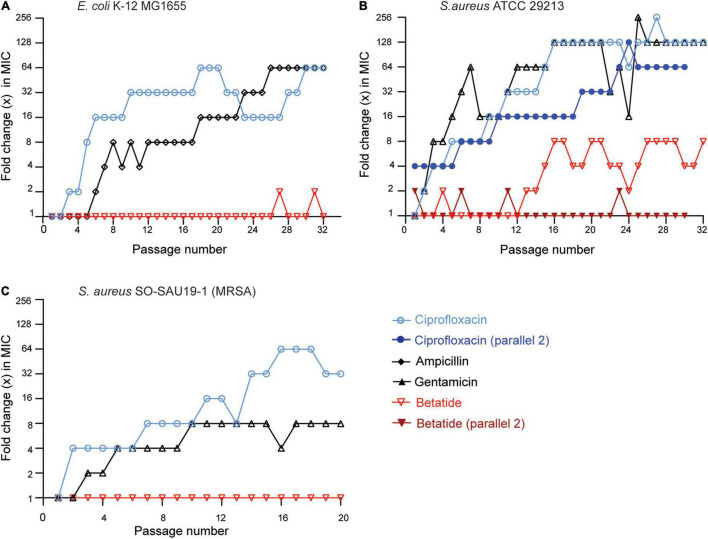
Low ability for bacteria to develop resistance against betatide after serial passages in media containing sub-MIC levels of betatide. Increase in MIC relative to day 1 in **(A)** *E. coli* K-12 MG1655, **(B)** *S. aureus* ATCC 29213, and **(C)** *S. aureus* SO-SAU19-1 (MRSA) after exposure to sub-MIC-level (0.25× and 0.5×) betatide and two reference antibiotics over 20–32 days. The bacteria were serial passaged every day with fresh media and treated with new doses of ciprofloxacin (blue circles), ampicillin (black diamonds) or gentamicin (black upright triangles), and betatide (red inverted triangles) to determine new MICs. Two independent experiments are shown for ciprofloxacin and betatide for *S. aureus* ATCC 29213 where parallel 2 is shown as darker shades of filled blue circles and filled red inverted triangles, respectively.

Long-time exposure to sub-MIC levels of antibiotics are known to increase TLS and induce resistance development (Kreuzer, 2013; Raeder et al., 2021). To directly examine the resistance development against betatide, we exposed *E. coli* and *S. aureus* (both MDR and reference strain) to sub-MIC and 1×–2× MIC levels of betatide through serial passage and measured MIC over 20–30 days. Compared to bacteria exposed to gentamicin, ampicillin, and ciprofloxacin, those exposed to betatide had a much lower capacity to develop resistance (Figure 3). In *E. coli*, betatide showed only a temporary 2× increase in MIC during these 32 days, compared to up to a 64× stable increase in MIC for ciprofloxacin and ampicillin (Figure 3A). *S. aureus* developed a higher increase in MIC toward all the treatments compared to *E. coli*: up to a 256× increase in MIC for ciprofloxacin and gentamicin, while only an 8× increase in MIC for betatide was detected in the first experiment (Figure 3B, parallel 1). As mutations are stochastic events, we repeated this experiment and found no detectable resistance development with betatide after 30 days (Figure 3B, parallel 2), while ciprofloxacin was more similar to parallel 1). Furthermore, an MDR strain of *S. aureus* (MRSA) did not show any signs of resistance development against betatide during 20 passages even though 8× and 32× increases in MIC against gentamicin and ciprofloxacin, respectively, were detected (Figure 3C). These experiments show reduced ability of the bacteria to develop resistance against betatide compared to commonly used antibiotics.

## Discussion

In this study, we show that the lead antibacterial APIM-peptide candidate, betatide, kills ESKAPE MDR variants and their corresponding reference strains with low MIC. Betatide is not cytotoxic to mammalian cells, does not hinder the epithelialization capacity of a human keratinocyte cell line, and kills both extracellular MRSE and intracellular *S. aureus* in infected cell cultures. More than just killing already resistant bacterial strains, no/little endogenous resistance against betatide is detected after long-time exposure in *E. coli* and *S. aureus*.

Many AMPs have been and are under development, but a majority of them are failing in clinical phases due to low stability, undesired immune responses, resistance development, and high cytotoxicity (Andersson et al., 2016; Magana et al., 2020). How betatide behaves *in vivo* needs to be further tested; however, data from the Phase I study of the similar anticancer APIM-peptide ATX-101 are promising as this peptide has a favorable toxicity profile (Lemech et al., 2021). The arginine-rich CPPs in APIM-peptides enable rapid uptake in bacteria, yeast, and mammalian cells, and the peptides are detected in all tissues examined upon intravenous infusion, including the brain (Muller et al., 2013; Olaisen et al., 2018; Søgaard et al., 2018). The preference for bacteria over mammalian cells for betatide compared to ATX-101 is increased by amidation of the C-termini (reduced MIC by ∼4×, unpublished data) and changes in the linker region between the APIM sequence and the CPP (reduced MIC by ∼2×, Table 2). Therefore, MICs for betatide are much lower than doses that affect mammalian cells. APIM-peptides kill bacteria rapidly, i.e., 1× MIC leads to 97% killing within 5 min (Raeder et al., 2021); thus, the short serum half-life of 15–30 min in humans found for the similar anticancer APIM-peptide ATX-101 (Lemech et al., 2021) may therefore be sufficient for good antibacterial *in vivo* activity. Short serum half-life may rather be an advantage as this lowers the chances of developing immunogenicity and affecting the normal microbiota if given intravenously and/or topically.

The relative increase in antibacterial and antimutagenic activity of betatide (peptide 2, R11) versus peptide 5 (R8) could be due to slightly more efficient uptake of the peptide in bacteria and thereby more APIM being available for interaction with the β-clamp. The reason for the improved activity by the GILQ-WRK-I linker compared to the W-KKKRK-I linker, used in the anticancer peptide and which is based on viral SV40 nuclear localization signal, is elusive. However, it could, as previously discussed (Nedal et al., 2020), involve altered β-clamp binding capacities of residues flanking the APIM-motif in addition to increased uptake and/or stability.

The likelihood of resistance development against a new antibacterial drug is a critical feature to consider prior to antibacterial drug development. Betatide targets the β-clamp and reduces mutation frequency in bacteria via inhibition of Pol V–β-clamp interaction at sub-MICs, and this was shown to reduce the bacteria’s ability to develop resistance against other antibiotics (Nedal et al., 2020). Here, we show that bacteria have a low ability to develop resistance against betatide. This, in combination with the low cytotoxicity of betatide in both HEK293 and HaCaT cells and the weak reduction of epithelization in HaCaT detected at concentrations more than 12× of what is required to abolish extracellular MRSE, warrants further examinations for topical use.

The resistance development to betatide is low, and we have so far not detected any cross-resistance with other antibiotics. One reason contributing to this could be that a mutation on the β-clamp that leads to reduced affinity for betatide is likely to also affect interactions with the polymerases and, thereby, affect both replication and TLS. This, in turn, can reduce the fitness of the bacteria, similar to what has been shown for another peptide targeting the β-clamp called griselimycin. Griselimycin-resistant mutants with mutations in the β-clamp were found to have considerably lower fitness than the wild-type (WT) *Mycobacterium smegmatis* (Kling et al., 2015). However, other resistance mechanisms not affecting the target, but the import of the peptide and/or degradation of the peptide, could lead to higher tolerance to betatide. It has recently been shown that the activation of the Cpx-envelope stress response system in *E. coli* can increase the tolerance toward antibacterial peptides and peptide nucleic acids (PNAs) containing arginine-rich CPPs by inhibiting the uptake (Frimodt-Moller et al., 2021). Betatide showed a 2× increase in MIC in Cpx mutant *E. coli* cells which had a constitutive active Cpx response (Frimodt-Moller et al., 2021). This resulted in reduced membrane potential in the inner membrane and thereby reduced uptake of the peptide. This tolerance mechanism is common for arginine-rich CPPs and other AMPs, such as LL37 (Audrain et al., 2013), and are therefore likely not due to the APIM-motif in betatide itself.

Some increase in resistance against betatide was detected in the first serial passage in the *S. aureus* reference strain, while not in the second serial passage or in the MDR strain of *S. aureus* (MRSA). Mutations are stochastic events, and the outcome of the mutations thus vary each time, and only mutations that increase tolerance while not compromising fitness would be detected in these experiments. However, all the experiments in *S. aureus*, together with the low resistance development in *E. coli*, indicate a low tendency to develop resistance against betatide compared to several commonly used antibiotics.

Betatide showed a broad-spectrum activity against multiple bacterial strains independent of resistance patterns toward other antibiotics. Since wound infections are not likely to be monocultures, broad antibacterial activity is a favorable trait for topical antibacterial treatments. Fusidic acid, an inhibitor of the bacterial elongator factor, is a bacteriostatic antibiotic with a novel target. It is commonly used topically to treat skin infections caused by both sensitive and MDR *S. aureus*. Because single mutations in multiple genes (e.g., *fus* A-C) can cause resistance to fusidic acid, the drug is often used in combination with other drugs, most commonly rifampicin (also bacteriostatic) (Fernandes, 2016). The additive effect observed when betatide was combined with fusidic acid (2×–8×) suggests that betatide could be an alternative broad-spectrum antibacterial drug for use in combination with fusidic acid.

## Conclusion

To summarize, betatide has antibacterial effects on both naive and resistant bacterial species (as the ESKAPE variants) without toxic effects on epithelialization. Its ability to impair resistance development toward other antibiotics and increase other antibiotics’ efficacy, in combination with the low ability of bacteria to develop resistance against betatide, warrants further examinations for use in ointments, creams, or gels for topical application.

## Data Availability Statement

The original contributions presented in the study are included in the article/Supplementary Material, further inquiries can be directed to the corresponding author/s.

## Author Contributions

AN, SR, and MO planned and initiated the study. AN, SR, CS, and MH performed the laboratory experiments and/or interpreted the results. AN, SR, CS, and MO wrote the manuscript. All authors contributed to the article and approved the submitted version.

## Conflict of Interest

The authors declare that the research was conducted in the absence of any commercial or financial relationships that could be construed as a potential conflict of interest.

## Publisher’s Note

All claims expressed in this article are solely those of the authors and do not necessarily represent those of their affiliated organizations, or those of the publisher, the editors and the reviewers. Any product that may be evaluated in this article, or claim that may be made by its manufacturer, is not guaranteed or endorsed by the publisher.

## Abbreviations

AMP: Antimicrobial peptide
AMR: Antimicrobial resistance
APIM: AlkB homolog 2 PCNA-interacting motif
CFU: Colony-forming unit
CPP: Cell-penetrating peptide
ESKAPE: *Enterococcus faecium* *Staphylococcus aureus* *Klebsiella pneumoniae* *Acinetobacter baumannii* *Pseudomonas aeruginosa* and *Enterobacter* species
MDR: Multidrug resistance
MIC: Minimal inhibitory concentration
MRSA: Methicillin-resistant *Staphylococcus aureus*
MRSE: Methicillin-resistant *Staphylococcus epidermidis*
PCNA: Proliferating cell nuclear antigen
TLS: Translesion synthesis.

## References

[B1] Andersson D. I. Hughes D. Kubicek-Sutherland J. Z. (2016). Mechanisms and consequences of bacterial resistance to antimicrobial peptides. *Drug Resist. Updat.* 26 43–57. 27180309 10.1016/j.drup.2016.04.002

[B2] Audrain B. Ferrières L. Zairi A. Soubigou G. Dobson C. Coppée J. Y. (2013). Induction of the Cpx envelope stress pathway contributes to *Escherichia coli* tolerance to antimicrobial peptides. *Appl. Environ. Microbiol.* 79 7770–7779. 10.1128/AEM.02593-13 24096425 PMC3837802

[B3] Beaber J. W. Hochhut B. Waldor M. K. (2004). SOS response promotes horizontal dissemination of antibiotic resistance genes. *Nature* 427 72–74. 10.1038/nature02241 14688795

[B4] Cockerill F. R. Wikler M. A. Alder J. Dudley M. N. Eliopoulos G. M. Ferraro M. J. (2012). *Methods For Dilution Antimicrobial Susceptibility Tests For Bacteria That Grow Aerobically: Approved Standard.* 9th ed. M07-A9. Wayne, PA: Clinical and Laboratory Standards Institute.

[B5] EUCAST (2021). *Breakpoint Tables For Interpretation Of Mics And Zone Diameters [Online]. The European Committee On Antimicrobial Susceptibility Testing.* Available online at: http://www.eucast.org [Accessed 11, 2021]

[B6] Fernandes P. (2016). Fusidic acid: a bacterial elongation factor inhibitor for the oral treatment of acute and chronic staphylococcal infections. *Cold Spring Harb. Perspect. Med.* 6:a025437. 10.1101/cshperspect.a025437 26729758 PMC4691801

[B7] Filius P. M. Gyssens I. C. (2002). Impact of increasing antimicrobial resistance on wound management. *Am. J. Clin. Dermatol.* 3 1–7. 10.2165/00128071-200203010-00001 11817964

[B8] Fraunholz M. Sinha B. (2012). Intracellular staphylococcus aureus: live-in and let die. *Front. Cell. Infect. Microbiol.* 2:43. 10.3389/fcimb.2012.00043 22919634 PMC3417557

[B9] Frimodt-Moller J. Koulouktsis A. Charbon G. Otterlei M. Nielsen P. E. Lobner-Olesen A. (2021). Activating the Cpx response induces tolerance to antisense PNA delivered by an arginine-rich peptide in *Escherichia coli*. *Mol. Ther. Nucleic Acids* 25 444–454. 10.1016/j.omtn.2021.06.009 34484867 PMC8403718

[B10] Futaki S. Suzuki T. Ohashi W. Yagami T. Tanaka S. Ueda K. (2001). Arginine-rich Peptides: an abundant source of membrane-permeable peptides having potential as carriers for intracellular protein delivery*. *J. Biol. Chem.* 276 5836–5840. 11084031 10.1074/jbc.M007540200

[B11] Gilljam K. M. Feyzi E. Aas P. A. Sousa M. M. Muller R. Vagbo C. B. (2009). Identification of a novel, widespread, and functionally important PCNA-binding motif. *J. Cell Biol.* 186 645–654. 10.1083/jcb.200903138 19736315 PMC2742182

[B12] Goodman M. F. (2002). Error-prone repair DNA polymerases in prokaryotes and eukaryotes. *Annu. Rev. Biochem.* 71 17–50. 10.1146/annurev.biochem.71.083101.124707 12045089

[B13] Iqbal Z. Seleem M. N. Hussain H. I. Huang L. Hao H. Yuan Z. (2016). Comparative virulence studies and transcriptome analysis of *Staphylococcus aureus* strains isolated from animals. *Sci. Rep.* 6 35442–35442. 10.1038/srep35442 27739497 PMC5064352

[B14] Kling A. Lukat P. Almeida D. V. Bauer A. Fontaine E. Sordello S. (2015). Antibiotics: targeting DnaN for tuberculosis therapy using novel griselimycins. *Science* 348 1106–1112. 10.1126/science.aaa4690 26045430

[B15] Kloos W. E. Musselwhite M. S. (1975). Distribution and persistence of *Staphylococcus* and *Micrococcus* species and other aerobic bacteria on human skin. *Appl. Microbiol.* 30 381–385. 10.1128/am.30.3.381-395.1975 810086 PMC187193

[B16] Kreuzer K. N. (2013). DNA damage responses in prokaryotes: regulating gene expression, modulating growth patterns, and manipulating replication forks. *Cold Spring Harb. Perspect. Biol.* 5:a012674. 10.1101/cshperspect.a012674 24097899 PMC3809575

[B17] Lemech C. R. Kichenadasse G. Marschner J. P. Alevizopoulos K. Otterlei M. Millward M. (2021). *Safety Profile And Disease Stabilization In Late Stage, Heavily Pretreated, Solid Tumor Patients In A First-In-Human (FIH) Study of ATX-101, A Drug Targeting Proliferating Cell Nuclear Antigen (PCNA).* Alexandria, VA: ASCO American Society of Clinical Oncology.

[B18] Longaker M. T. Harrison M. R. Langer J. C. Crombleholme T. M. Verrier E. D. Spendlove R. (1989). Studies in fetal wound healing: II. A fetal environment accelerates fibroblast migration in vitro. *J. Pediatr. Surg.* 24 793–798. 10.1016/s0022-3468(89)80539-1 2769548

[B19] Magana M. Pushpanathan M. Santos A. L. Leanse L. Fernandez M. Ioannidis A. (2020). The value of antimicrobial peptides in the age of resistance. *Lancet Infect. Dis.* 20 e216–e230. 32653070 10.1016/S1473-3099(20)30327-3

[B20] Massey R. C. Horsburgh M. J. Lina G. Höök M. Recker M. (2006). The evolution and maintenance of virulence in *Staphylococcus aureus*: a role for host-to-host transmission? *Nat. Rev. Microbiol.* 4 953–958. 10.1038/nrmicro1551 17109032

[B21] Merrikh H. Kohli R. M. (2020). Targeting evolution to inhibit antibiotic resistance. *FEBS J.* 287 4341–4353. 10.1111/febs.15370 32434280 PMC7578009

[B22] Muller R. Misund K. Holien T. Bachke S. Gilljam K. M. Vatsveen T. K. (2013). Targeting proliferating cell nuclear antigen and its protein interactions induces apoptosis in multiple myeloma cells. *PLoS One* 8:e70430. 10.1371/journal.pone.0070430 23936203 PMC3729839

[B23] Nedal A. Raeder S. B. Dalhus B. Helgesen E. Forstrom R. J. Lindland K. (2020). Peptides containing the PCNA interacting motif APIM bind to the beta-clamp and inhibit bacterial growth and mutagenesis. *Nucleic Acids Res.* 48 5540–5554. 10.1093/nar/gkaa278 32347931 PMC7261172

[B24] Olaisen C. Kvitvang H. F. N. Lee S. Almaas E. Bruheim P. Drablos F. (2018). The role of PCNA as a scaffold protein in cellular signaling is functionally conserved between yeast and humans. *FEBS Open Bio* 8 1135–1145. 10.1002/2211-5463.12442 29988559 PMC6026702

[B25] Otto M. (2009). Staphylococcus epidermidis–the ‘accidental’ pathogen. *Nat. Rev. Microbiol.* 7 555–567. 10.1038/nrmicro2182 19609257 PMC2807625

[B26] Pastar I. Stojadinovic O. Yin N. C. Ramirez H. Nusbaum A. G. Sawaya A. (2014). Epithelialization in wound healing: a comprehensive review. *Adv. Wound Care (New Rochelle)* 3 445–464. 10.1089/wound.2013.0473 25032064 PMC4086220

[B27] Pham P. Rangarajan S. Woodgate R. Goodman M. F. (2001). Roles of DNA polymerases V and II in SOS-induced error-prone and error-free repair in *Escherichia coli*. *Proc. Natl. Acad. Sci. U.S.A.* 98 8350–8354. 10.1073/pnas.111007198 11459974 PMC37442

[B28] Raeder S. B. Sandbakken E. T. Nepal A. Loseth K. Bergh K. Witso E. (2021). Novel peptides targeting the beta-clamp rapidly kill planktonic and biofilm staphylococcus epidermidis both in vitro and in vivo. *Front. Microbiol.* 12:631557. 10.3389/fmicb.2021.631557 33815313 PMC8009970

[B29] Rousselle P. Braye F. Dayan G. (2019). Re-epithelialization of adult skin wounds: cellular mechanisms and therapeutic strategies. *Adv. Drug Deliv. Rev.* 146 344–365. 10.1016/j.addr.2018.06.019 29981800

[B30] Silverman J. A. Oliver N. Andrew T. Li T. (2001). Resistance studies with daptomycin. *Antimicrob. Agents Chemother.* 45 1799–1802. 11353628 10.1128/AAC.45.6.1799-1802.2001 PMC90548

[B31] Søgaard C. K. Blindheim A. Røst L. M. Petrović V. Nepal A. Bachke S. (2018). “Two hits – one stone”; increased efficacy of cisplatin-based therapies by targeting PCNA’s role in both DNA repair and cellular signaling. *Oncotarget* 9 32448–32465. 10.18632/oncotarget.25963 30197755 PMC6126690

[B32] Tuchscherr L. Medina E. Hussain M. Völker W. Heitmann V. Niemann S. (2011). Staphylococcus aureus phenotype switching: an effective bacterial strategy to escape host immune response and establish a chronic infection. *EMBO Mol. Med.* 3 129–141. 10.1002/emmm.201000115 21268281 PMC3395110

[B33] Tulloch L. G. (1954). Nasal carriage in staphylococcal skin infections. *Br. Med. J.* 2 912–913. 10.1136/bmj.2.4893.912 13199342 PMC2079316

[B34] Walter M. N. Wright K. T. Fuller H. R. Macneil S. Johnson W. E. (2010). Mesenchymal stem cell-conditioned medium accelerates skin wound healing: an in vitro study of fibroblast and keratinocyte scratch assays. *Exp. Cell Res.* 316 1271–1281. 10.1016/j.yexcr.2010.02.026 20206158

[B35] World Health Organization (2015). *Global Action Plan On Antimicrobial Resistance [Online].* Geneva: World Health Organization.

[B36] World Health Organization (2017). *WHO Publishes List Of Bacteria For Which New Antibiotics Are Urgently Needed [Online].* Geneva: World Health Organization.

[B37] World Health Organization (2018). *Antimicrobial Resistance [Online].* Geneva: World Health Organization.

[B38] Yakimov A. Bakhlanova I. Baitin D. (2021). Targeting evolution of antibiotic resistance by SOS response inhibition. *Comput. Struct. Biotechnol. J.* 19 777–783. 10.1016/j.csbj.2021.01.003 33552448 PMC7843400

