# Supplementary material for: Broad-Spectrum Antibacterial Peptide Kills Extracellular and Intracellular Bacteria Without Affecting Epithelialization
Source: Front Microbiol. 2021 Nov 26;12:764451. doi: 10.3389/fmicb.2021.764451 (PMC8661032; doi:10.3389/fmicb.2021.764451)
Supplement: Supplementary file 1 [file Data_Sheet_1.PDF]

Broad-spectrum antibacterial peptide kills extra- and intracellular bacteria without affecting epithelialization

Supplementary Material

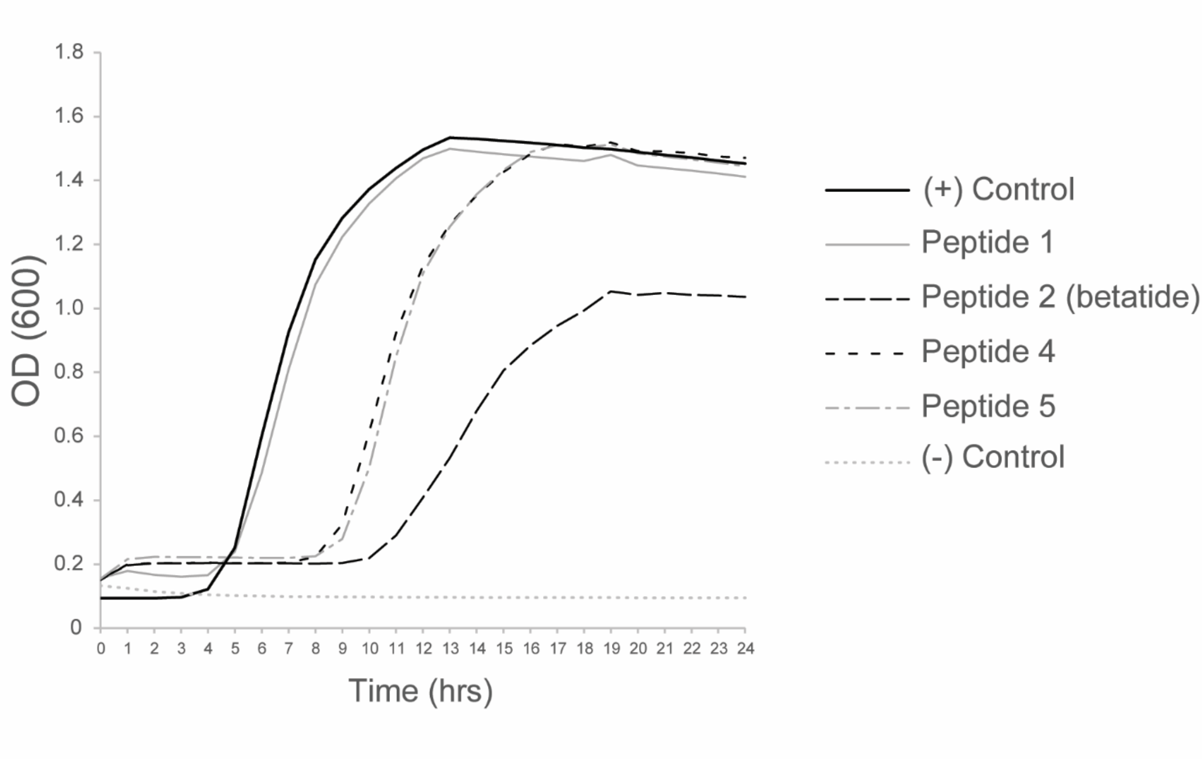

Supplementary Figure S1 (related to Table 2): Peptide 2 (betatide) efficiently inhibits growth of *E. coli*. Growth of *E. coli* in LB medium over 24 hours (Optical Density (OD) 600 nm) after addition of APIM-peptide variants 1-5 (60 µg/mL) at time t=0 (see Table 2). Negative (-) control is no bacteria added, positive (+) control is untreated bacteria.

Bacteria treated with peptide 2 (betatide, long black dashes) had lowest OD after 8 hours, indicating highest inhibition of growth. Peptide 1 (gray filled line) is the least efficient compared to untreated control (black filled line). Negative control (-, grey dashed line) showed no growth.

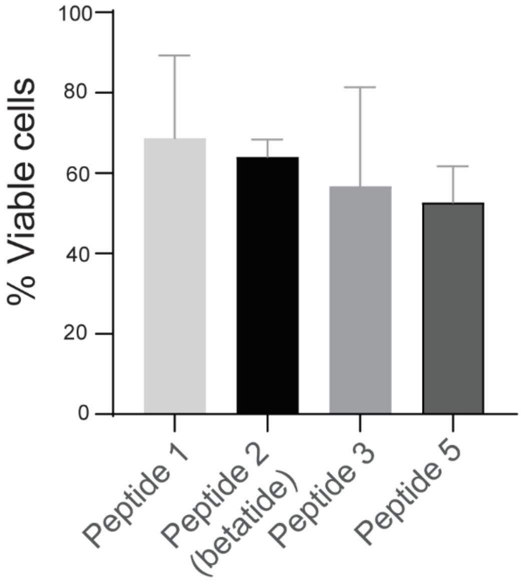

Supplementary Figure S2 (related to Table 2): Peptide 2 (betatide) does not reduce the growth of HEK293 differently from the other peptides.

The bars show percentage (%) viable cells of HEK293 after treatment with APIM-peptides (24 µg/mL) accessed by MTT-assay after 72 hours relative to untreated control. Data for 3 biological replicates with mean ± SD is shown.

None of the peptides show significant reduction in growth compared to untreated control. At 48 µg/mL all peptides were toxic, while 12 µg/mL gave same results as 24 µg/mL (data not shown).

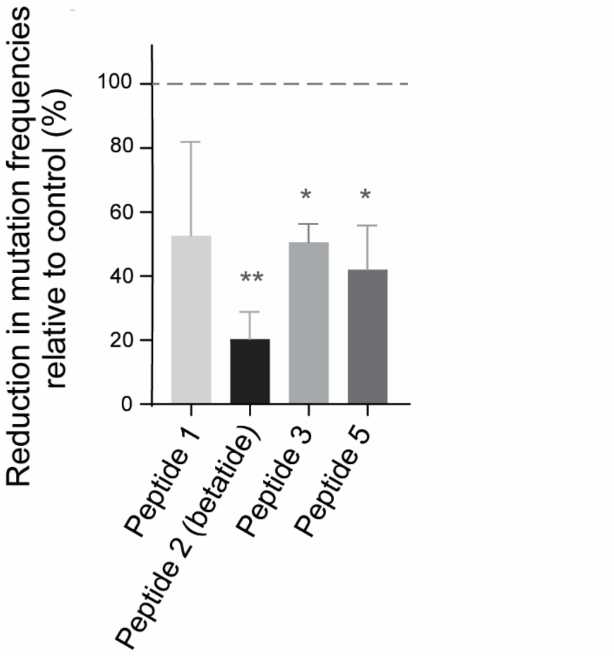

Supplementary Figure S3 (related to Table 2): Peptide 2 (betatide) reduces UV-induced mutagenesis in *E. coli* more efficiently than the other peptide variants.

Increase in mutation frequencies after treatment with UV-C (20 mJ/cm^2) relative to spontaneous mutations frequencies using the Rif^R assay were determined. This increase is set to 100% for untreated control, and % reduction after addition of peptides 1-5 (20 µg/mL) are shown. Mean ± SD from three independent experiments. * p<0.05, ** p<0.005

Peptide 2, 3 and 5 all significantly reduced the mutations frequencies relative to control, and peptide 2 (betatide) showed the largest reduction.
